# Translation and validation of the Dutch version of the Effective Consumer Scale (EC-17)

**DOI:** 10.1007/s11136-012-0162-2

**Published:** 2012-03-28

**Authors:** Peter M. ten Klooster, Erik Taal, Liseth Siemons, Johanna C. M. Oostveen, Etelka J. Harmsen, Peter S. Tugwell, Tamara Rader, Anne Lyddiatt, Mart A. F. J. van de Laar

**Affiliations:** 1 Department of Psychology, Health and Technology, Arthritis Centre Twente, University of Twente, PO Box 217, 7500 AE Enschede, The Netherlands; 2 Department of Rheumatology, Ziekenhuisgroep Twente, PO Box 7600, Almelo, The Netherlands; 3 Centre for Global Health, Faculty of Medicine, University of Ottawa, 1 Stewart Street, Ottawa, ON K1N 6N5 Canada; 4 Department of Rheumatology, Arthritis Centre Twente, Medisch Spectrum Twente, PO Box 50000, 7500 KA Enschede, The Netherlands

**Keywords:** Arthritis, Consumer participation, Psychometrics, Rasch analysis

## Abstract

**Purpose:**

The Effective Consumer Scale (EC-17) measures the skills of musculoskeletal patients in managing their own healthcare. The objectives of this study were to translate the EC-17 into Dutch and to further evaluate its psychometric properties.

**Methods:**

The EC-17 was translated and cognitively pretested following cross-cultural adaptation guidelines. Two hundred and thirty-eight outpatients (52 % response rate) with osteoarthritis or fibromyalgia completed the EC-17 along with other validated measures. Three weeks later, 101 patients completed the EC-17 again.

**Results:**

Confirmatory factor analysis supported the unidimensional structure of the scale. The items adequately fit the Rasch model and only one item demonstrated differential item functioning. Person reliability was high (0.92), but item difficulty levels tended to cluster around the middle of the scale, and measurement precision was highest for moderate and lower levels of skills. The scale demonstrated adequate test–retest reliability (ICC = 0.71), and correlations with other measures were largely as expected.

**Conclusion:**

The results supported the validity and reliability of the Dutch version of the EC-17, but suggest that the scale is best targeted at patients with relatively low levels of skills. Future studies should further examine its sensitivity to change in a clinical trial specifically aimed at improving effective consumer skills.

## Introduction

Self-management interventions are aimed at providing patients with the necessary knowledge, skills, and confidence to effectively manage their condition themselves. The effectiveness of such interventions is typically evaluated by a wide range of clinical severity measures, self-reported symptoms, and presumed psychological mediators such as self-efficacy [1–3]. To date, however, there is no agreement on the actual set of attributes that are important to managing and participating in healthcare and on how to measure these [4]. This makes it difficult to evaluate direct effects on patient skills and to compare the results of various interventions.

To address this issue, the 17-item Effective Consumer Scale (EC-17) was recently developed based on extensive literature reviews, expert and patient interviews and pilot testing [5]. A follow-up study explored its construct validity and responsiveness in participants in the arthritis self-management program (ASMP) [6]. Results showed that the EC-17 addressed skills and behaviours not covered by other relevant scales such as the Health Education Impact Questionnaire [7], Patient Activation Measure [8], and Arthritis Self-Efficacy Scale [9], including identifying quality information and negotiation with health professionals [6]. Moreover, although the ASMP was not tailored to all behaviours measured by the EC-17, the scale was modestly sensitive to change [6]. A similar study examining the Norwegian EC-17 also showed that the scale was easy to complete, internally consistent, reproducible, valid, and moderately responsive to change [10]. The aim of this study was to cross-culturally adapt the EC-17 for use in Dutch patients with musculoskeletal conditions and to evaluate its psychometric properties.

## Materials and methods

### Cross-cultural translation

Cross-cultural adaptation followed established forward–backward translation procedures [11]. The prefinal EC-17 was cognitively pretested in five patient research partners (four female, age range 29–74 years) with different rheumatic conditions. Pretests were carried out using the three-step test interview method [12]. Based on the results, small wording changes were made in six items (e.g., ‘arrange’ instead of ‘organise’), one response option (‘generally’ instead of ‘usually’), and the instructions (expanded with an explanation of the term ‘management’).

### Psychometric evaluation

#### Participants

A survey was sent in October 2010 to a random sample of 404 patients with osteoarthritis (OA) and 58 patients with fibromyalgia (FM) that had visited the outpatient rheumatology clinic in the preceding year. Two hundred and fifty-three (54.8 %) patients returned a completed survey. The first 120 patients willing to complete the scale a second time were sent a follow-up questionnaire, which was completed by 101 (84.2 %) patients after a median (IQR) time of 20 (18–24) days.

#### Measures

The EC-17 measures knowledge, attitudes, and behaviours about self-management skills using 17 items with 5-point Likert-type scales (“never” to “always”) [5]. Item scores are summed when ≥14 items are completed and converted to range from 0 to 100, where 100 is the best possible score.

Additionally, patients completed the 5-item Perceived Efficacy in Patient–Physician Interactions scale (PEPPI-5; *α* = 0.90) [13, 14], the 12-item Dutch General Self-Efficacy Scale (GSES; *α* = 0.80) [15, 16], the 4-item support from family and friends subscale from the Arthritis Impact Measurement Scales 2 (AIMS2; *α* = 0.91) [17, 18], and the 36-Item Short Form Health Survey (SF-36v2) [19, 20], from which the physical and mental component summary (PCS and MCS) scores were calculated [21]. Pain in the last week was measured on an 11-point numerical rating scale (NRS) from 0 (‘no pain’) to 10 (‘unbearable pain’).

#### Data analysis

Fifteen patients had >3 missing values on the EC-17 and were excluded from further analyses (final response rate 51.5 %). Remaining missing values were low, with a maximum of five (2.1 %) for items 10 and 16, and were imputed with their median values.

Unidimensionality of the EC-17 was tested using robust maximum likelihood confirmatory factor analysis (CFA) [22]. Non-normed (NNFI) and comparative fit (CFI) indexes ≥0.95 and standardized root mean square residual (SRMR) and root mean square error of approximation (RMSEA) ≤0.08 and 0.06, respectively, were considered indicative of good fit [23, 24].

Additionally, Rasch partial credit model analyses were performed [25]. Conservative infit values between 0.87 and 1.13 and outfit values between 0.61 and 1.39 were considered to indicate acceptable item fit [26]. Items with residual correlations >0.30 were considered locally dependent [27, 28]. Differential item functioning (DIF) was evaluated across sex, age, and disease duration and considered present when the difference between the item calibrations was statistically significant and >0.5 logits [25, 29]. Person reliability ≥0.70 and ≥0.85 was considered adequate for group-level and individual comparisons, respectively [28]. The person-item map and test information function were examined for mistargeting and local measurement precision [30].

Reproducibility was assessed by intraclass correlation (ICC, type A,1) [31] and considered adequate for group-level and individual measurements over time when ≥0.70 and ≥0.90, respectively [32].

For convergent and discriminant validity, it was hypothesized that an adequate measure of perceived health-management skills should be strongly correlated with perceived effectiveness in patient–physician interaction, which is an important aspect of general health-management skills, and moderately correlated with the conceptually related construct of general self-efficacy and social support [33–35]. Finally, a moderate correlation with psychosocial health (SF-36 MCS) and weak correlations with physical health (SF-36 PCS) and pain were expected [36].

## Results

### Patient characteristics

Patient characteristics are reported in Table 1. There were no significant differences with respect to age or sex between the respondents and non-respondents. FM patients differed significantly from OA patients on several socio-demographic variables and scored worse on all scales, except the GSES and SF-36 PCS.

**Table 1:** Patient characteristics

Characteristic	OA *n* = 209	FM *n* = 29	*P*	Total *N* = 238
Age, years	62.6 ± 10.1	42.4 ± 14.4	<0.001	60.1 ± 12.6
Sex, female	169 (80.9 %)	25 (86.2 %)	N.S.	194 (81.5 %)
Disease duration, years	10.9 ± 10.9	10.2 ± 10.1	N.S.	10.8 ± 10.8
Ethnicity, Dutch	198 (94.7 %)	27 (93.1 %)	N.S.	225 (94.5 %)
Self-management training, yes	13 (6.2 %)	9 (31.0 %)	<0.001	22 (9.2 %)
Marital status				
Not married/not living together	4 (1.9 %)	6 (20.7 %)	<0.001	10 (4.2 %)
Married/living together	158 (75.6 %)	20 (69.0 %)		178 (74.8 %)
Widowed/divorced	45 (21.6 %)	3 (10.3 %)		48 (20.2 %)
Education				
Low	127 (60.7 %)	11 (37.9 %)	0.070	138 (58.0 %)
Medium	44 (21.1 %)	14 (48.3 %)		58 (24.4 %)
High	35 (16.8 %)	4 (13.8 %)		39 (16.4 %)
Occupational status				
Full-time employed	25 (12.0 %)	2 (6.9 %)	<0.001	27 (11.3 %)
Part-time employed	49 (23.4 %)	12 (41.4 %)		61 (25.6 %)
Homemaker	51 (24.4 %)	5 (17.2 %)		56 (23.5 %)
School	1 (0.5 %)	4 (13.8 %)		5 (2.1 %)
Unemployed/disabled/retired	80 (38.3 %)	6 (20.6 %)		86 (36.1 %)
EC-17 (range 0–100)	68.9 ± 16.3	62.3 ± 14.1	0.040	68.1 ± 16.1
PEPPI-5 (range 5–25)	18.7 ± 4.3	16.8 ± 3.1	0.005	18.5 ± 4.2
GSES (range 12–60)	42.8 ± 6.3	42.9 ± 7.5	N.S.	42.8 ± 6.5
AIMS2 Support (range 0–10)	3.7 ± 2.5	4.7 ± 2.4	0.039	3.8 ± 2.5
SF-36 PCS (range 0–100)	36.0 ± 9.2	35.7 ± 6.4	N.S.	35.9 ± 8.9
SF-36 MCS (range 0–100)	49.0 ± 10.6	43.7 ± 12.0	0.015	48.4 ± 10.9
NRS Pain (range 0–10)	5.7 ± 2.0	7.1 ± 1.2	<0.001	5.8 ± 2.0

Values are mean ± SD or number (%). *OA* osteoarthritis, *FM* fibromyalgia, *PEPPI*-5 perceived efficacy in patient–physician interactions scale, *EC*-17 effective consumer scale, *GSES* general self-efficacy scale, *AIMS*2 arthritis impact measurement scales 2, *SF*-36 medical outcomes study 36-item short form, *PCS* physical component summary, *MCS* mental component summary, *NRS* numerical rating scale

### Distributional properties

**Fig. 1:**
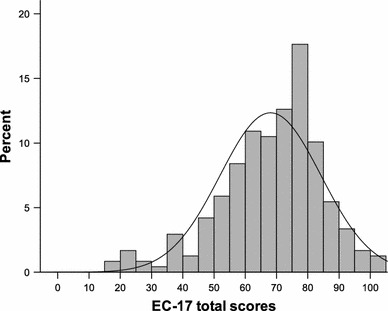
Distribution of EC-17 total scores

Total scores on the EC-17 showed a near-normal distribution (Kolmogorov–Smirnov, *P* = 0.058) with skewness and kurtosis values of −0.72 and 0.74, respectively (Fig. 1). Floor and ceiling effects were absent, with no patients scoring zero and only three patients (1.3 %) scoring 100.

### Unidimensionality

With the exception of RMSEA, the one-factor model showed a good fit (SBχ^2^(119) = 488.70, NNFI = 0.96, CFI = 0.96, SRMR = 0.08, RMSEA (90 % CI) = 0.11 (0.10–0.12). Standardized factor loadings were high for all items (Table 2).

**Table 2:** Standardized factor loadings and Rasch item parameters and fit statistics of the EC-17 ordered by difficulty level

Item	Factor loading	Item difficulty in logits (SE)	Infit MNSQ	Outfit MNSQ	Absolute DIF in logits		
					Sex^a^	Age^b^	Disease duration^c^
16. I can negotiate with the healthcare system about what to do to manage my disease	0.80	0.67 (0.09)	0.98	1.01	−0.14	0.15	−0.35
13. I feel a sense of control over my disease	0.73	0.56 (0.10)	1.09	1.15	−0.25	0.28	0.21
10. I am able to play the role I want to in my healthcare team	0.71	0.36 (0.10)	1.06	1.12	0.11	−0.03	0.00
1. I know who can help me judge the quality of the information I receive about my disease	0.66	0.36 (0.09)	1.19	1.33	−0.09	−0.29	−0.05
15. I can negotiate with others about what we need to do to manage my disease	0.85	0.30 (0.10)	0.74	0.75	−0.03	0.06	0.00
17. I can organise my life to act on decisions about how to manage my disease	0.82	0.22 (0.10)	0.86	0.80	−0.15	0.34	−0.09
11. I know who to work with to meet my health needs	0.76	0.19 (0.10)	0.91	0.88	−0.25	0.14	0.00
12. I can be assertive to get what I need to meet my health needs (for example, information and treatments)	0.83	0.18 (0.10)	0.78	0.74	0.10	0.11	−0.13
6. I can set realistic goals about the management of my disease	0.67	−0.01 (0.11)	1.09	1.03	0.05	−0.23	−0.02
4. I can be clear about what is important in my life when I make decisions about my disease	0.68	−0.08 (0.12)	1.01	1.03	0.16	−0.20	0.18
9. I have built an open and trusting relationship, based on mutual respect, with my healthcare providers	0.70	−0.15 (0.09)	1.13	1.20	−0.31	0.42	0.05
7. I can express my concerns well to healthcare providers	0.74	−0.26 (0.10)	0.97	1.01	0.35	0.24	−0.22
5. I can weigh the pros and cons of a decision about my disease	0.70	−0.35 (0.11)	1.02	0.98	0.31	−0.18	0.23
8. I know how to ask good questions about my health and my disease	0.77	−0.37 (0.11)	0.92	0.92	0.05	−0.31	−0.17
14. I feel confident in making decisions about my health	0.76	−0.39 (0.11)	0.97	0.94	0.00	0.33	−0.11
3. I know how to adapt general health information to my own situation	0.68	−0.57 (0.11)	1.00	1.03	0.09	−0.48	0.46
2. I understand the information I receive about my disease	0.64	−0.68 (0.11)	1.17	1.09	0.29	−0.80*	0.21

Higher positive logit scores indicate more difficult items

^a^Male versus female; ^b^ median split ≤59 years versus >59 years; ^c^ median split ≤6 years versus >6 years

* Significant at Bonferroni adjusted level of *P* < 0.001

### Rasch measurement properties

The EC-17 adequately fit the Rasch model. Five items showed infit values slightly outside the range of 0.87–1.13, and no items showed poor outfit (Table 2). Residual correlations revealed some redundancy or multidimensionality, as demonstrated by 4 pairs of items showing positive (*r’s* between 0.33 and 0.40) and 4 showing negative local dependence (*r’s* between −0.31 and −0.32). No items showed DIF across sex and disease duration and only one item across age.

**Fig. 2:**
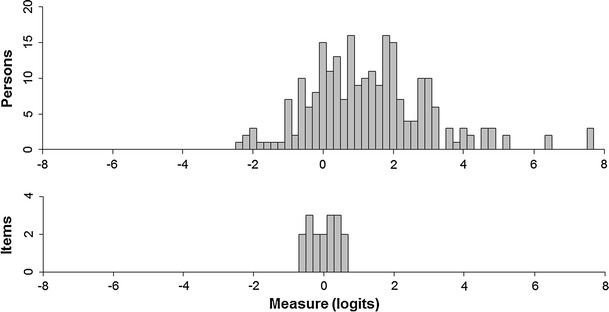
Distribution of person abilities and item difficulties across the scale. Higher positive logit scores indicate better self-management skills and more difficult items. Mean person ability = 1.25 (SD = 1.74); mean item difficulty = 0.00 (SD = 0.39)

Internal consistency was sufficiently high (person reliability = 0.92) for individual-level comparisons. Item difficulty estimates ranged from 0.67 to −0.68 logits and tended to cluster around the middle of the scale, with a large proportion of patients with relatively high skills not being covered by any individual item (Fig. 2). The mean logit score for patients was 1.25, indicating that the sample as a whole was located at a higher ability level than the mean item difficulty.

**Fig. 3:**
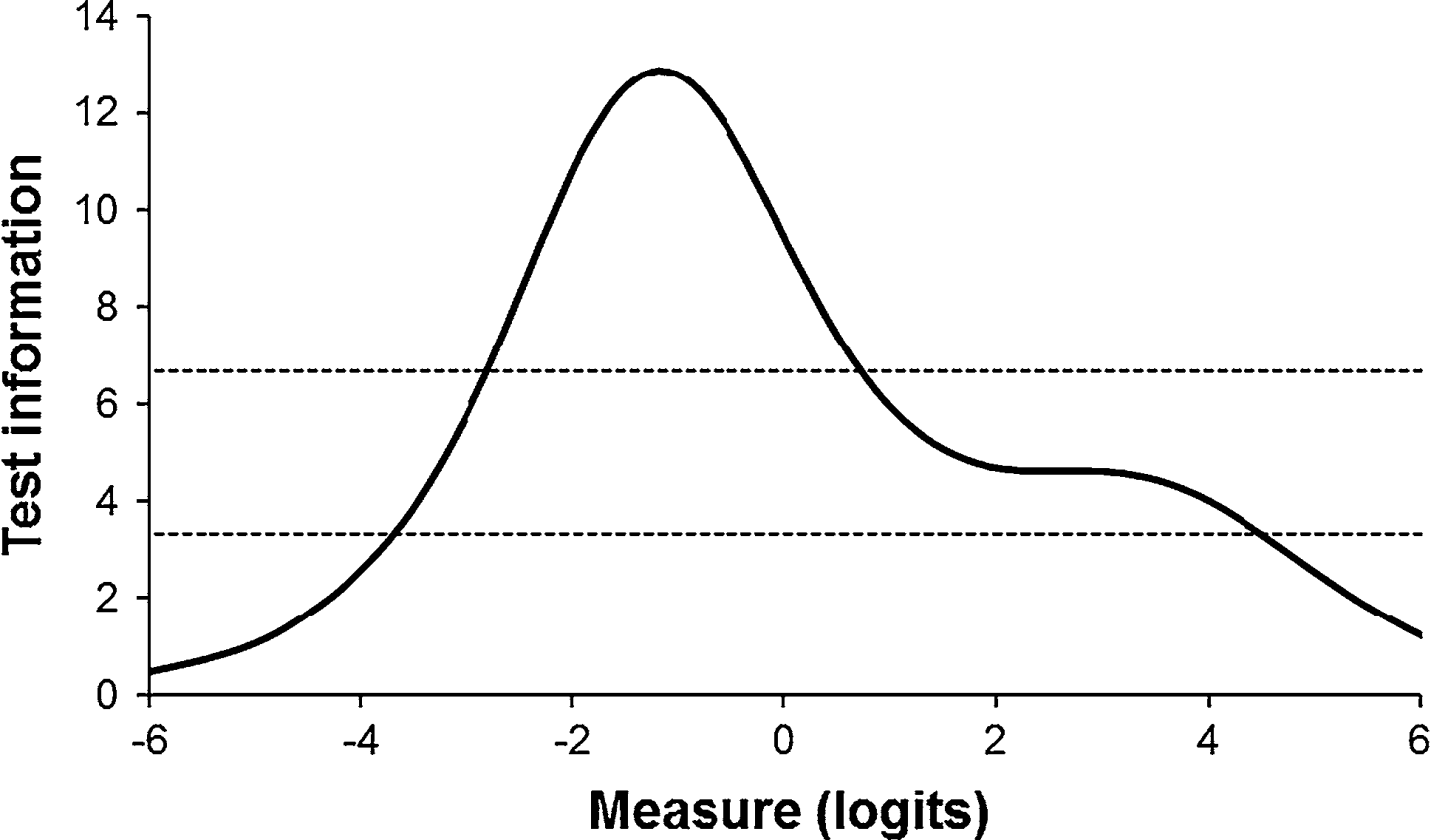
Test information curve of the EC-17 in relation to the Rasch score. Higher positive logit scores indicate better self-management skills and attributes. Test information values of 3.33 and 6.67 (*dotted lines*) correspond to a reliability of 0.70 and 0.85, respectively. Logit values of −6, 0, and 6 correspond to approximate total scores on the EC-17 of 1, 59, and 98, respectively

The information curve (Fig. 3) was peaked at lower levels of the underlying trait, indicating that patients with skills below the mean are measured with more precision than individuals with better skills. Measurement precision was sufficient for group-level analyses across a wide range of the underlying trait, but adequate for individual-level comparisons in persons with moderate and lower levels of self-management skills only.

### Test–retest reliability

With an ICC of 0.71 (95 % CI: 0.60–0.80), test–retest reliability of the scale was adequate for group-level comparisons.

### External construct validity

As expected, the EC-17 correlated strongly with perceived efficacy in patient–physician interactions, moderately with social support and psychosocial aspects of health, and weakly with physical aspects of health and pain (Table 3). The association with general self-efficacy was just below the cut-off value for moderate correlation.

**Table 3:** Pearson correlations between the EC-17 and other measures in total sample

Measure	Expected *r*	Observed *r*
PEPPI-5	0.5–1.0	0.55**
GSES	0.3–0.5	0.26**
AIMS2 Support	0.3–0.5	−0.34**
SF-36 MCS	0.3–0.5	0.39**
SF-36 PCS	0.0–0.3	0.14*
NRS Pain	0.0–0.3	−0.21**

*EC*-17 17-item effective consumer scale, *PEPPI*-5 5-item perceived efficacy in patient–physician interactions scale, *GSES* general self-efficacy scale, *AIMS*2 arthritis impact measurement scales 2, *SF*-36 medical outcomes study 36-item short form, *MCS* mental component summary, *PCS* physical component summary, *NRS* numerical rating scale

* *P* < 0.05, ** *P* < 0.01

## Discussion

EC-17 scores were normally distributed, and the results of both CFA and Rasch analysis supported the unidimensionality of the scale, indicating that item scores can be summed to create a single total score. The latter is in accordance with previous studies that used principal component analyses [6, 10].

The high internal consistency of the EC-17 corresponds to the ability to discriminate between 3 and 4 distinct levels of skills [25]. However, measurement precision was not equally high across the underlying trait. On a group level, the EC-17 had sufficient precision across a wide range of scores. However, it was adequate for individual-level comparisons only in persons with moderate and lower levels of skills. Although it may be desirable to have a measure that specifically targets patients with lower skills, this also suggests that the EC-17 may lack discriminatory power in patients with relatively high levels of skills. Since a sample size of approximately 240 persons has been shown to provide accurate estimates of item and person locations in Rasch analyses, even for measures with poor targeting, these results are likely to be quite robust [37]. However, no other studies have used Rasch analyses for the current 17 items, and this finding should be further investigated in other populations.

Test–retest reliability was adequate, but lower than previously found [10]. It is possible that we used a more strict ICC model [38] or that the time interval was too long to assure that no inter-individual variation occurred.

Finally, with the exception of general self-efficacy, all hypothesized correlations were confirmed, supporting the convergent and discriminant validity of the scale.

Given the relatively low response rate and the differences in both demographic and clinical characteristics between the OA and FM patients, the current findings should be interpreted with some caution and be cross-validated in other samples of musculoskeletal patients.

In conclusion, this study suggests that Dutch EC-17 is a valid and reliable measure of effective health consumer skills in patients with musculoskeletal conditions. Future studies should further examine its sensitivity to change in a clinical trial specifically aimed at improving the skills and behaviours deemed necessary for effective consumers, before the scale can be fully endorsed as an outcome measure for evaluating self-management interventions.

## Abbreviations

CFA: Confirmatory factor analysis
DIF: Differential item functioning
EC-17: 17-item Effective Consumer Scale
FM: Fibromyalgia
IRT: Item response theory
OA: Osteoarthritis
RA: Rheumatoid arthritis
